# The implications of different approaches to define AT(N) in Alzheimer disease

**DOI:** 10.1212/WNL.0000000000009485

**Published:** 2020-05-26

**Authors:** Niklas Mattsson-Carlgren, Antoine Leuzy, Shorena Janelidze, Sebastian Palmqvist, Erik Stomrud, Olof Strandberg, Ruben Smith, Oskar Hansson

**Affiliations:** From the Clinical Memory Research Unit, Department of Clinical Sciences (N.M.-C., A.L., S.J., S.P., E.S., O.S., R.S., O.H.), and Wallenberg Centre for Molecular Medicine (N.M.-C.), Lund University, Malmö; and Department of Neurology (N.M.-C., S.P., R.S.) and Memory Clinic (E.S., O.H.), Skåne University Hospital, Lund, Sweden.

## Abstract

**Objective:**

To compare different β-amyloid (Aβ), tau, and neurodegeneration (AT[N]) variants within the Swedish BioFINDER studies.

**Methods:**

A total of 490 participants were classified into AT(N) groups. These include 53 cognitively unimpaired (CU) and 48 cognitively impaired (CI) participants (14 mild cognitive impairment [MCI] and 34 Alzheimer disease [AD] dementia) from BioFINDER-1 and 389 participants from BioFINDER-2 (245 CU and 144 CI [138 MCI and 6 AD dementia]). Biomarkers for A were CSF Aβ_42_ and amyloid-PET ([^18^F]flutemetamol); for T, CSF phosphorylated tau (p-tau) and tau PET ([^18^F]flortaucipir); and for (N), hippocampal volume, temporal cortical thickness, and CSF neurofilament light (NfL). Binarization of biomarkers was achieved using cutoffs defined in other cohorts. The relationship between different AT(N) combinations and cognitive trajectories (longitudinal Mini-Mental State Examination scores) was examined using linear mixed modeling and coefficient of variation.

**Results:**

Among CU participants, A−T−(N)− or A+T−(N)− variants were most common. However, more T+ cases were seen using p-tau than tau PET. Among CI participants, A+T+(N)+ was more common; however, more (N)+ cases were seen for MRI measures relative to CSF NfL. Tau PET best predicted longitudinal cognitive decline in CI and p-tau in CU participants. Among CI participants, continuous T (especially tau PET) and (N) measures improved the prediction of cognitive decline compared to binary measures.

**Conclusions:**

Our findings show that different AT(N) variants are not interchangeable, and that optimal variants differ by clinical stage. In some cases, dichotomizing biomarkers may result in loss of important prognostic information.


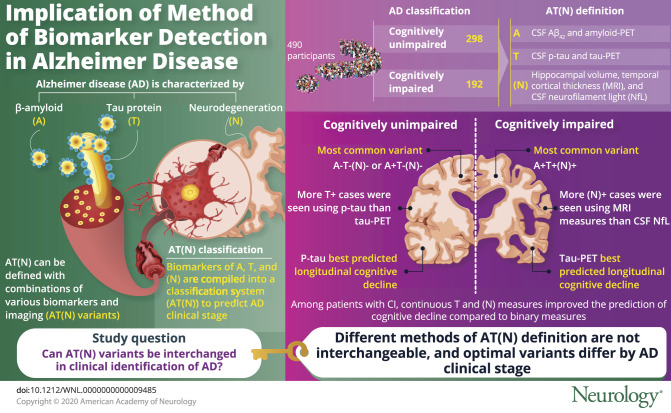


Alzheimer disease (AD) is characterized by accumulation of β-amyloid (Aβ) and tau, atrophy, and cognitive decline.^1^ The different brain changes can be monitored by CSF biomarkers, MRI, and PET. The National Institute on Aging and Alzheimer's Association has proposed a framework for research^2^ based on the idea that biological processes start before symptoms in AD. The framework suggests that biomarkers of Aβ (A), tau (T), and neurodegeneration (N) can be compiled into a classification system (AT[N]). The framework is purposely agnostic to the details of operationalization, and it is unknown how different variants of AT(N) (using different biomarkers) compare. We therefore tested a variety of AT(N) approaches, with different combinations of biomarkers, which were compared in terms of prevalence in cognitively unimpaired (CU) and cognitively impaired (CI) participants and to predict cognitive decline. We hypothesized that different AT(N) variants would have considerable differences in category prevalence and that the prediction of longitudinal cognitive decline would vary both by AT(N) variant and predictor data type (binary vs continuous).

## Methods

### Participants

We included individuals from the Swedish BioFINDER studies (BioFINDER-1, clinical trial NCT01208675; BioFINDER-2, clinical trial NCT03174938). BioFINDER-1 included 53 CU and 48 CI participants (14 mild cognitive impairment [MCI] and 34 AD dementia). CU individuals were aged ≥60 years and did not have MCI or dementia.^2–4^ Exclusion criteria included presence of objective cognitive impairment, severe somatic disease, and current alcohol or substance abuse. Patients with MCI fulfilled the DSM-5 criteria for minor cognitive impairment.^5^ Patients with AD dementia fulfilled the DSM-5 criteria for major cognitive impairment due to AD.^5^ Patients with MCI and patients with AD had low CSF Aβ_42_/Aβ_40_ levels (<0.10). Exclusion criteria were cognitive impairment that could better be accounted for by another non-neurodegenerative condition, severe somatic disease, and current alcohol or substance abuse. Longitudinal cognitive data were available in all individuals from BioFINDER-1. To validate cross-sectional results, we also included 389 participants from the Swedish BioFINDER-2 study (245 CU and 144 CI participants) (table e-1, doi.org/10.5061/dryad.p5hqbzkkx).

### Standard protocol approvals, registrations, and patient consents

All participants gave written informed consent. Ethical approval was given by the Regional Ethical Committee of Lund University. Approval for PET imaging was obtained from the Swedish Medical Products Agency and the local Radiation Safety Committee at Skåne University Hospital.

### CSF biomarkers

Procedures for CSF collection, processing, and storage have been described.^6^ CSF Aβ (Aβ_42_), tau phosphorylated at Thr181 (p-tau), and neurofilament light (NfL) were quantified using ELISAs (Aβ_42_ and p-tau: BioFINDER-1, EUROIMMUN AG, Lübeck, Germany; BioFINDER-2, INNOTEST, Fujirebio, Ghent, Belgium; NfL: Uman Diagnostics, Umeå, Sweden).^7^ CSF analyses were performed in accordance with the Alzheimer's Association Flow Chart for CSF biomarkers.^8^

### Imaging acquisition and processing

T1-weighted MRI was performed on a 3T Siemens Tim Trio scanner (Siemens Medical Solutions, Erlangen, Germany) using a sagittal magnetization-prepared rapid gradient echo sequence.^9^ FreeSurfer (v. 5.3, surfer.nmr.mgh.harvard.edu/) was used to extract hippocampal volume (adjusted for intracranial volume) and a cortical thickness measure within a meta–region of interest (ROI) encompassing temporal regions with known susceptibility in AD (mean thickness in the bilateral entorhinal, inferior temporal, middle temporal, and fusiform cortices, adjusted for surface area).^10^

Amyloid and tau imaging were performed using [^18^F]flutemetamol and [^18^F]flortaucipir PET, respectively, as described elsewhere.^11,12^ Briefly, dynamic (list-mode) studies were performed over the 90- to 100-minute postinjection interval for amyloid PET and 80- to 100-minute interval for tau PET. In BioFINDER-2, tau PET was performed using [^18^F]RO948,^13^ with dynamic (list-mode) studies performed over the 70- to 90-minute interval.

Target ROIs were selected on the basis of previously published findings: a global neocortical composite for amyloid PET^6^ and, for tau PET, the inferior temporal cortex (ITC, as a measure of relatively early tangle pathology)^14^ and a meta-ROI corresponding to widespread relatively late stage neocortical tangle pathology (Braak V/VI) (table e-2, doi.org/10.5061/dryad.p5hqbzkkx).^15^ Standardized uptake value ratio (SUVR) images were created normalizing to a composite region (whole cerebellum, pons/brainstem, and eroded cortical white matter) for [^18^F]flutemetamol and the inferior cerebellar cortex for [^18^F]flortaucipir and [^18^F]RO948.

### Cognition

Global cognition was assessed using longitudinal Mini-Mental State Examination (MMSE) (tested at an average [median] of 4 time points [interquartile range (IQR) 3–5] over a median time span of 4.7 years [IQR 8 months to 4.2 years]).

### AT(N) definitions

AT(N) biomarkers included CSF Aβ_42_ (A1), amyloid PET ([^18^F]flutemetamol PET neocortical SUVR) (A2), CSF p-tau (T1), tau PET ([^18^F]flortaucipir) SUVR within the ITC (T2) and composite Braak V/VI region (T3), hippocampal volume (N1), cortical thickness within the temporal meta-ROI (N2), and CSF NfL (N3). Binarization of biomarkers (+/−, normal/abnormal) was done using cutoffs established using Gaussian mixture modeling (GMM) in BioFINDER-1 for amyloid PET, CSF, and MRI-based measures, and in Alzheimer's Disease Neuroimaging Initiative (ADNI) for tau PET (table-e3 and figure e-1, doi.org/10.5061/dryad.p5hqbzkkx). The only exception was hippocampal volume, which was not suitable for GMM due to a clear unimodal distribution. We therefore used a Youden index–derived cutoff instead (Aβ-positive MCI vs Aβ-negative CU controls, with Aβ status defined by the CSF Aβ_42_/Aβ_40_ ratio). This method, along with the mean ±2 SD from Aβ-negative CU controls (+2 SD for amyloid PET, CSF p-tau, tau PET, and CSF NfL; −2 SD for CSF Aβ_42_, hippocampal volume, and temporal lobe thickness), were also used for all biomarkers as a sensitivity analysis to examine the effect of cutoff selection.

These 4 terms are used throughout the article: (1) “AT(N) variant,” a specific combination of A, T, and (N) (e.g., A1T1(N)2 is a variant); (2) “AT(N) category,” a combined biomarker profile in an AT(N) variant (e.g., A1+T1−[N]2− is a category); (3) “AT(N) component,” refers to the A, T, or (N) position; and (4) “AT(N) biomarker,” refers to a specific measure for a component (e.g., CSF Aβ_42_ is the A biomarker in the variant A1T1[N]2).

### Statistical analyses

Demographics and biomarkers were compared between diagnostic groups using Kruskal-Wallis test or Fisher exact test. Associations between continuous biomarkers were summarized using Spearman rank correlation (ρ), Cohen kappa statistic (κ), and percentage agreement (concordance). Prevalence estimates for AT(N) categories were calculated in CU and CI participants with confidence intervals generated using bootstrap resampling (n = 1,000). Prevalence findings for AT(N) categories were further examined in a separate cohort (the Swedish BioFINDER-2 study). The relationship between AT(N) categories and cognitive trajectories (longitudinal MMSE) was examined using linear mixed-effects (LME) modeling, with subject-specific intercepts and slopes, adjusted for age, sex, and education, with a restricted cubic spline term for time, to account for nonlinear trajectories. LME models with different AT(N) variants as predictors were compared by marginal *R*^2^. LME analyses were performed using both binary and continuous biomarker predictors. All analyses were performed in R, v.3.5.3 (R Foundation for Statistical Computing, R-project.org/), with significance set at *p* < 0.05, 2-tailed.

### Data availability

Anonymized study data for the primary analyses presented in this report are available on request from any qualified investigator for purposes of replicating the results.

## Results

### Study participants

Demographics are presented in table 1. No significant differences were found between CU and CI participants in sex, education, or prevalence of *APOE* ε4. There was a trend that CU participants were older in BioFINDER-1, and they were significantly younger than CI in BioFINDER-2. CI participants had lower MMSE, Aβ_42_, hippocampal volume, and temporal meta-ROI cortical thickness, and higher p-tau, amyloid and tau PET SUVRs, and NfL. As CSF NfL has been shown to be associated with age^16^ we divided participants into younger and older using a median split and compared CSF NfL levels between resulting groups. We found that there was no significant difference in NfL levels between groups (*p* = 0.365); this was also the case when dividing the participants using age 65 as a cutpoint (*p* = 0.590), and when running the same analyses in BioFINDER-2. Participant numbers and median NfL values for these 2 comparisons are summarized in table e-4 (doi.org/10.5061/dryad.p5hqbzkkx). On the basis of these analyses, the prevalence of (N)+ using NfL is unlikely to vary by age in the present cohort.

**Table 1:**
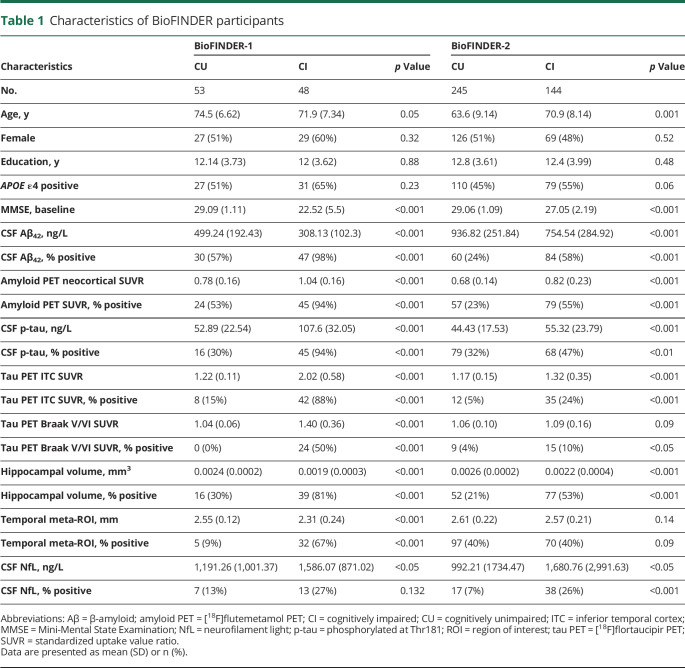
Characteristics of BioFINDER participants

### Biomarker relationships

Cutoffs were defined as CSF Aβ_42_ < 510.4 ng/L (A1), amyloid PET >0.743 SUVR (A2), p-tau >60.2 ng/L (T1), ITC tau PET >1.29 SUVR (T2), Braak V/VI tau PET >1.32 SUVR (T3), hippocampal volume (adjusted for intracranial volume, i.e., hippocampal volume/intracranial volume) <0.00215 (N1), temporal meta-ROI thickness <2.38 mm (N2), and CSF NfL >1850 ng/L (N3). Similar cutoffs were obtained using the Youden index and mean ± 2 SD from Aβ-negative CU controls, except for CSF Aβ_42_, where the mean −2 SD resulted in a more conservative cutoff (table e-3, doi.org/10.5061/dryad.p5hqbzkkx).

**Figure 1:**
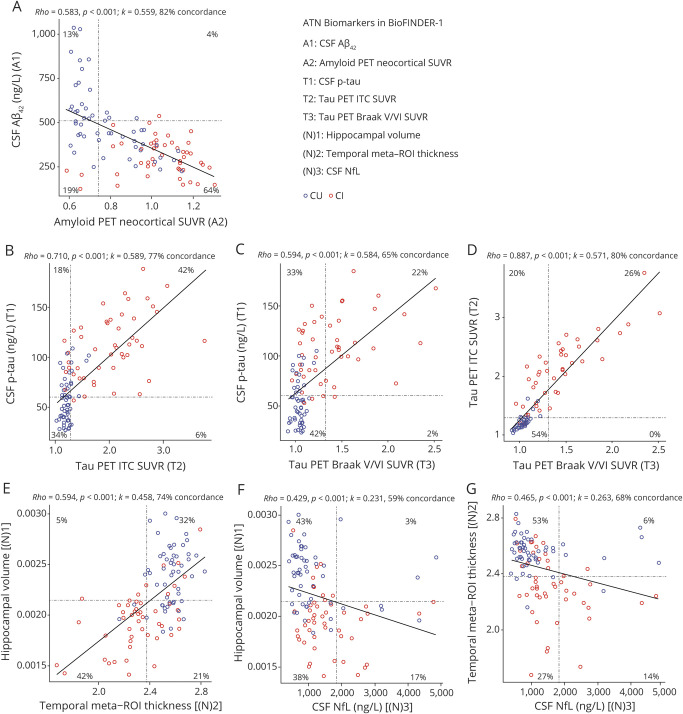
Pairwise scatterplots for A, T, and (N) variables in BioFINDER-1 Scatterplots show the association between continuous measures for amyloid (A), tau (B–D), and (N) (E–G) biomarkers. Dashed lines indicate cutoff points. Spearman correlations (ρ) with *p* values, Cohen kappa statistic (κ), and concordance (percentage showing both biomarkers positive or negative) are shown at the top of each panel. For A and T comparisons, the upper right and lower left quadrants indicate concordance positive (+/+) and negative (−/−), respectively. For the comparisons (N)1 vs (N)2, the lower left and upper right quadrants indicate concordance positive and negative, respectively. For the 2 remaining (N) comparisons, concordant positive is in the lower right quadrant while concordant negative is in the upper left. Percentage figures across quadrants indicate distribution (percentage-wise) of participants. Aβ = β-amyloid; AT(N) = β-amyloid, tau, and neurodegeneration classification system; CI = cognitively impaired; CU = cognitively unimpaired; ITC = inferior temporal cortex; NfL = neurofilament light; p-tau = tau phosphorylated at Thr181; ROI = region of interest; SUVR = standardized uptake value ratio.

Continuous biomarker measures were correlated: CSF Aβ_42_ (A1) vs amyloid PET (A2) (ρ = −0.583; figure 1A), p-tau (T1) vs ITC tau PET (T2) (ρ = 0.710) and Braak V/VI (T3) (ρ = 0.594), as well as between the 2 tau PET-based measures (ρ = 0.887; figure 1, B–D); hippocampal volume (N)1 vs temporal meta-ROI cortical thickness (N)2 (ρ = 0.594) and vs NfL (N)3 (ρ = −0.429); and temporal meta-ROI cortical thickness vs NfL (ρ = −0.465; all *p* < 0.001; figure 1, E–G).

Using binary data, moderate agreement was seen between amyloid biomarkers (κ = 0.559, concordance = 82%), between p-tau and tau PET SUVR in the ITC (κ = 0.589; concordance = 77%) and Braak V/VI meta-ROI (κ = 0.584, concordance = 65%), and between the 2 tau PET measures (κ = 0.571, concordance = 80%). For measures of neurodegeneration, the imaging measures had slightly better concordance with each other (hippocampal volume and temporal meta-ROI cortical thickness, κ = 0.458, concordance = 74%) than with NfL (hippocampal volume and NfL, κ = 0.231, concordance = 59%; temporal meta-ROI cortical thickness and NfL, κ = 0.263, concordance = 68%).

### Prevalence measures in CU participants

**Figure 2:**
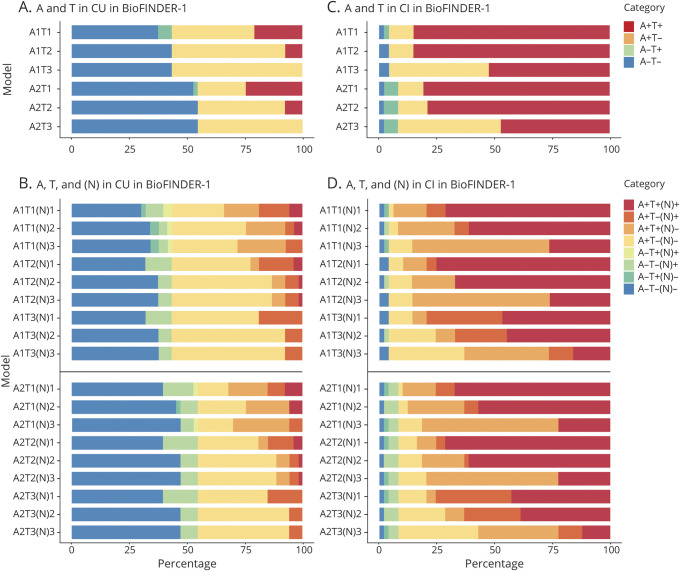
Prevalence of different AT(N) categories in different AT(N) variants among cognitively unimpaired (CU) and cognitively impaired (CI) participants in BioFINDER-1 Prevalence is reported without (A,C) and with (B, D) consideration for the (N) component. CSF Aβ_42_ (A1); amyloid PET neocortical standardized uptake value ratio (SUVR) (A2); CSF tau phosphorylated at Thr181 (T1); tau PET inferior temporal cortex SUVR (T2); tau PET Braak V/VI SUVR (T3); hippocampal volume, adjusted for intracranial volume ([N]1); cortical thickness with a temporal meta–region of interest ([N]2); CSF neurofilament light ([N]3). AT(N) = β-amyloid, tau, and neurodegeneration classification system.

**Figure 3:**
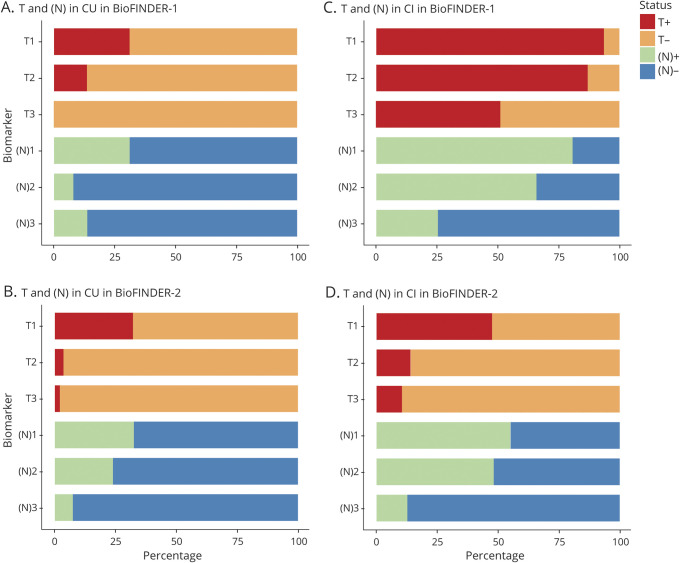
Prevalence of T and (N) positivity across cognitively unimpaired (CU) and cognitively impaired (CI) participants in BioFINDER-1 and BioFINDER-2 Prevalence estimates for CU participants are shown in (A) (BioFINDER, n = 101: 53 CU and 48 CI participants) and (B) (BioFINDER-2, n = 389: 245 CU and 144 CI participants) and for CI participants in (C) (BioFINDER-1) and (D) (BioFINDER-2). AT(N) = β-amyloid, tau, and neurodegeneration classification system.

Prevalences for AT(N) categories based on GMM cutoffs are summarized for CU and CI participants in figure 2 and tables e-5 and e-6 (doi.org/10.5061/dryad.p5hqbzkkx). When examining A and T only among CU participants (figure 2A and table e-7, doi.org/10.5061/dryad.p5hqbzkkx), most were negative for both when using amyloid PET (A2), with a higher percentage showing isolated amyloid positivity when using CSF Aβ_42_ (A1). Positivity in both A and T was highest when using CSF p-tau (T1) (22% and 26% were T+ in the A1+ and A2+ groups, respectively). The prevalence of T+ was much less when using tau PET SUVR in ITC (T2) (9% were T+ in the A1+ and A2+ groups) or tau PET Braak V/VI SUVR (T3) (0% T+). These results show that CSF p-tau (T1) results in a much higher prevalence of T+ compared to tau PET in CU participants. Similar findings (i.e., higher prevalence of A+ using CSF Aβ_42_ and lower prevalence of T+ using tau PET than CSF p-tau) were found using Youden and mean ±2 SD based cutoffs (tables e-8–e-11, doi.org/10.5061/dryad.p5hqbzkkx) and using GMM in the BioFINDER-2 cohort (figure 3 and tables e-12 and e-13, doi.org/10.5061/dryad.p5hqbzkkx). The pattern for tau biomarkers also held in both cohorts when using tau PET in a very early ROI (the entorhinal cortex: tables e-14 and e-15, doi.org/10.5061/dryad.p5hqbzkkx).

When adding (N) biomarkers (figure 2B), A−T−(N)− (range 38% [A1T1(N)1; 95% confidence interval, 24.5%–50.9%] to 53% [A2T3(N)2; 95% confidence interval, 35.9%–64.2%]) or A+T−(N)− (range 13% [A2T1(N)1; 95% confidence interval, 5.7%–22.6%] to 43% [A1T3(N)2; 95% confidence interval, 30.2%–56.6%]) were the most common categories (table e-5, doi.org/10.5061/dryad.p5hqbzkkx). Although all 8 possible categories were represented in some AT(N) variants, several categories were absent or had very low frequencies (A−T+[N]−, A−T+[N]+, A+T+[N]+, and, when using tau PET, A+T+[N]−). Among the different biomarkers for (N), hippocampal volume (N)1 resulted in more (N)+ cases than when using cortical thickness in the temporal lobe meta-ROI ([N]2) and CSF NfL ([N]3) (figure 2B). This finding was also observed when using Youden and mean ± 2D cutoffs (tables e-16 through e-19, doi.org/10.5061/dryad.p5hqbzkkx) and in the BioFINDER-2 cohort (figure 3; table e-20, doi.org/10.5061/dryad.p5hqbzkkx). Overall, however, fewer A+ cases were seen among CU participants in BioFINDER-2 as compared to BioFINDER-1 (table e-21, doi.org/10.5061/dryad.p5hqbzkkx).

### Prevalence measures in CI participants

Positivity for both A and T components was the main finding when using only biomarkers for A and T in CI (figure 2C and tables e-22–e-26, doi.org/10.5061/dryad.p5hqbzkkx). However, when using the Braak V/VI ROI for tau PET (T3), A+T−(N)− was seen in approximately half of participants, because T3+ was relatively rare. Using A, T, and (N) components (figure 2D), A+T+(N)+ was the predominant finding for most AT(N) variants, ranging from 12% (A2T3[N]3; 95% confidence interval, 4.1%–22.5%) to 76% (A1T2[N]1; 95% confidence interval, 63.3%–87.8%). When CSF NfL (N)3 was used, A+T+(N)− was the most common category, because CSF NfL (N)3 was often normal ([N]+ in only 27% of CI participants) compared to MRI-based measures ([N]1, 78%; [N]2, 65%). Again, several categories were absent or had low frequencies (A+T−[N]−, A−T+[N]−, A−T−[N]+, A−T+[N]+) (table e-27, doi.org/10.5061/dryad.p5hqbzkkx). Similar findings were observed when using Youden and mean ± 2 SD based cutoffs (tables e-28–e-30, doi.org/10.5061/dryad.p5hqbzkkx). A comparable pattern of prevalence findings was also seen for CI participants in BioFINDER-2 (i.e., fewer T+ cases using the Braak V/VI ROI for tau PET [T3] and more [N]+ cases using hippocampal volume ([N]1) and temporal cortex thickness ([N]2) as compared to CSF NfL ([N]3) (figure 3 and table e-31, doi.org/10.5061/dryad.p5hqbzkkx). The prevalence of the A+T+(N)+ category was lower across AT(N) variants in BioFINDER-2 as compared to BioFINDER-1 (table e-21, doi.org/10.5061/dryad.p5hqbzkkx).

### Longitudinal cognition

**Figure 4:**
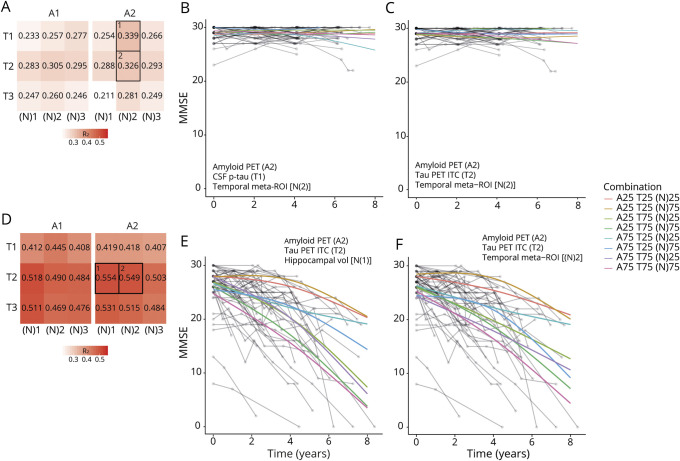
AT(N) variants and longitudinal cognition (A, D) *R*^2^ for different AT(N) variants to predict longitudinal Mini-Mental State Examination (MMSE) for cognitively unimpaired (CU) and cognitively impaired (CI) participants, respectively (divided by A biomarkers). The selected models in (B,C) and (E, F) are the top 2 best for CU and CI participants, respectively; 25 and 75 refer to 25th and 75th quartiles, where higher indicates a more abnormal biomarker. All CI participants were positive using CSF Aβ_42_ in ratio with Aβ_40._ AT(N) = β-amyloid, tau, and neurodegeneration classification system. ITC = inferior temporal cortex; p-tau = phosphorylated at Thr181; ROI = region of interest.

Overall findings for longitudinal cognition are summarized in figure 4 (continuous data) and tables e-32 and e-33 (binary data) and tables e-34 and e-35 (continuous data) (doi.org/10.5061/dryad.p5hqbzkkx). Using continuous predictors, the AT(N) variant combining amyloid PET, p-tau, and temporal cortical thickness (A2T1[N]2) best captured changes in cognition in CU participants (*R*^2^ = 0.339) (table 2). Comparison of models using pairwise analysis of variance (ANOVA) (i.e., A2T1[N]2 vs T1[N]2, *p* = 0.018; A2T1[N]2 vs A2T1, *p* = 0.0006; and A2T2[N]1 vs A2[N]2, *p* = 0.004) showed that all included variables (i.e., A2, T1, and [N]2) contributed significantly to the prediction of cognitive decline. The 2 best models in CU included amyloid PET (A2) and temporal meta-ROI cortical thickness (N)2 (figure 4, A–C). By contrast, in CI, the AT(N) variant combining amyloid PET, tau PET ITC, and hippocampal volume (A2T2[N]1) was the best model (*R*^2^ = 0.554) (figure 4, D and E). In this model, pairwise ANOVA (A2T1[N]2 vs T1[N]2, *p* = 0.018; A2T1[N]2 vs A2T1, *p* = 0.0006; and A2T2[N]1 vs A2[N]2, *p* = 0.004) again showed that all 3 biomarkers contributed significantly. Among CI, the 2 models that best predicted cognitive decline included amyloid PET (A2) and tau PET ITC SUVR (T2) (figure 4, D–F).

**Table 2:**
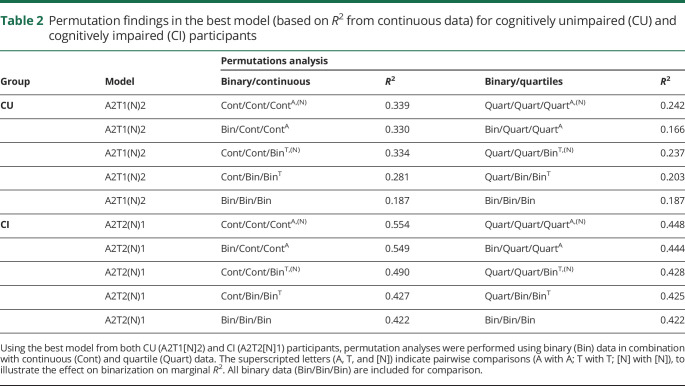
Permutation findings in the best model (based on *R*^2^ from continuous data) for cognitively unimpaired (CU) and cognitively impaired (CI) participants

We next tested the effect of switching from continuous to binary data for the biomarkers. This led to A2T2(N)2 and A2T2(N)3 having the highest *R*^2^ for CU (0.292) and CI (0.463) participants, respectively. Overlap among the best 5 models from continuous and binary data was partial (60% among CU participants, 40% among CI participants); the range of *R*^2^ values across models was narrow for CI participants (range 0.357–0.463) and somewhat broader for CU participants (range 0.090–0.292). Among the top 5 best models, the shift from continuous to binary data resulted in an average decrease in *R*^2^ of 0.101 for CU participants and 0.111 for CI participants.

### Mixing binary and continuous data in AT(N)

Due to the observed differences between continuous and binary data, we tested mixing binary and continuous predictors. To reduce the number of comparisons, we performed these analyses only on the top 5 best (based on *R*^2^ from continuous data) models for MMSE in CU and CI. Findings are presented in table 2 (model 1) and in tables e-36 and e-37 (doi.org/10.5061/dryad.p5hqbzkkx) (models 2–5). Among CU participants, the differences in *R*^2^ between binary and continuous predictors were small for CSF Aβ_42_ (A1) (e.g., binary/continuous, A1T2[N]2, *R*^2^ = 0.303–0.308) but greater for amyloid PET (A2) (e.g., A2T2[N]2, *R*^2^ = 0.307–0.316). A larger difference in *R*^2^ was seen between binary and continuous data for p-tau (T1) (e.g., A2T1[N]2, *R*^2^ = 0.281–0.325) and CSF NfL ([N]3) (e.g., A1T2[N]3, *R*^2^ = 0.268–0.295), but not for tau PET or MRI measures of neurodegeneration in CU (e.g., A2T2[N]3, *R*^2^ = 0.306–0.311 for T; *R*^2^ = 0.300–0.306 for [N]). Among CI participants, little difference in *R*^2^ was observed for binary and continuous A biomarkers, suggesting that the dichotomization captured most relevant variability in the A biomarkers (e.g., A1T2[N]1, *R*^2^ = 0.511–0.515; A2T2[N]1, *R*^2^ = 0.549–0.554). However, higher *R*^2^ values were consistently seen when using continuous measures of T (e.g., A2T2[N]1, *R*^2^ = 0.427–0.490) and (N) (e.g., A2T2[N]1, *R*^2^ = 0.490–0.554), suggesting that valuable information was lost when dichotomizing T and (N) biomarkers in CI. To explore alternatives to binary vs continuous biomarker groups, a further analysis was performed combining binary and quartile predictors. This confirmed the patterns observed when combining binary and continuous data (table 2 and tables e-36 and e-37, doi.org/10.5061/dryad.p5hqbzkkx).

## Discussion

This study of several different AT(N) variants confirmed our hypotheses that different operationalizations of the AT(N) system have strong effects on category prevalence and predictions of future cognitive decline. Our first main finding was that different AT(N) variants, using different biomarkers for A, T, and (N), give considerable differences in the classification of CU and CI participants. For example, a much larger proportion of CU participants are classified as T+ using CSF p-tau than when using tau PET, and a larger proportion of CI participants are classified as (N)+ using MRI measures than when using CSF NfL. The second main finding was that different AT(N) variants have different associations with longitudinal cognition, with differences between CU and CI (CSF p-tau was more influential in CU participants, and tau PET in CI participants). The third main finding was that using binary (rather than continuous) biomarker data affects prediction of longitudinal cognition, and some biomarkers were more suitable than others for dichotomization. Taken together, these results show that different AT(N) variants result in both different classifications of individuals at baseline and differences in the prognosis of future cognitive decline. This has implications for how to use and interpret AT(N) in research studies, clinical trial design, and potentially also in clinical practice. For example, prevention trials focusing on the very early (preclinical) stages of AD may benefit from using CSF p-tau to define T+, but prevention trials in the clinical stage (or assessments in clinical practice) may benefit from using tau PET instead.

The AT(N) system includes both fluid and imaging biomarkers,^2,17^ and our findings show that these modalities are not always interchangeable, especially not in all clinical disease stages. This is in line with a recent study that found that concordance between AT(N) biomarkers varied across CU and CI groups, and appeared to be stage dependent.^18^ Although the overall prevalence of amyloid positivity among CU individuals has been shown to be similar between cohorts assessed with CSF Aβ_42_ or amyloid PET,^19^ our finding (from a direct comparison within the same participants) that the proportion of CU participants defined as A+ was higher when using CSF Aβ_42_ suggests that amyloid imaging alone may underestimate early amyloid positivity. This is in line with previous findings in other cohorts that CSF Aβ_42_ may capture early stage amyloid pathology before amyloid PET.^20,21^ Similar to A, tau positivity was much higher among CU participants using p-tau, and a greater difference was seen between CSF p-tau and tau PET. This may be due to a temporal lag between these measures or differences in variance in both the negative range (likely greater for CSF) and the positive range (likely greater for PET).^22^ The discrepancy between prevalence when using NfL and MRI-based measures of (N) was a somewhat unexpected finding, given that both CSF NfL and MRI-based measures are altered in MCI and AD.^23,24^ However, following initial increases, the change in NfL may slow during the symptomatic course of the disease.^25,26^ Group differences in NfL (CI > CU), combined with similar levels across MCI and AD and the fact that NfL was only a significant predictor of longitudinal MMSE among CU participants, supports this scenario.

In order to verify the prevalence findings across AT(N) categories, we repeated prevalence calculations in a separate and larger cohort (the BioFINDER-2 cohort). Similar to findings in BioFINDER-1, we found higher prevalence of T+ using CSF p-tau and (N)+ using hippocampal volume among CU participants. Further, we found fewer T+ cases using tau PET in the Braak V/VI region and more (N)+ cases using MRI measures, as compared to CSF NfL, among CI participants. These findings were validated despite the fact that the BioFINDER-2 cohort used other technologies for tau PET and CSF biomarkers than BioFINDER-1. These findings therefore strongly support the idea that biomarker interchangeability across A, T, and (N) categories is likely to vary according to biomarker selection and clinical stage of the disease. Some differences were observed between cohorts, however, in terms of the overall prevalence of certain AT(N) variants, due to the BioFINDER-2 cohort having an overall lower frequency of A+ participants and a CI group comprising very few dementia cases as a result of the study having only recently been launched. Finally, while the cutoffs identified using the Youden index and mean ± 2 SD from Aβ-negative controls as part of the sensitivity analysis were similar to those using GMM, some differences were noted for A1 and (N)3. Though the 3 methods used are well-established, this finding highlights the optimization of cutoff selection (i.e., for predicting different outcomes based on the research question at hand) as an important area for future studies.

Most AT(N) variants with stronger associations with longitudinal cognition had [^18^F]flutemetamol as the A component, consistent with findings that amyloid PET is a later marker than CSF Aβ_42_.^6,20^ In line with findings that p-tau behaves as an early disease state marker in AD^9,27^ (i.e., reflecting the intensity of these disease process, and changing already prior to symptom onset), while tau PET behaves more as a stage marker (i.e., increasing steadily with disease progression),^28^ the best model for predicting cognition in CU (A2T1[N]2) included p-tau as the T variable, while tau PET was clearly superior in CI individuals. This is consistent with a temporal ordering of tau biomarkers where p-tau is most dynamic during the preclinical phase of AD, and tau PET in the clinical phase. Even among CU individuals, however, tau PET was associated with cognitive decline. Though few CU participants had elevated tau PET values, half had ITC SUVR values 20%–30% above the reference region, in line with the known association between cognition and tau PET signal within this region.^14^ The distribution of (N) biomarkers across the best models (CU: 3/5 [N]2, 2/5 [N]3; CI: 3/5 [N]1, 2/5 [N]2) may reflect variability in the patterns of neurodegeneration biomarkers across the AD continuum,^29^ with CSF NfL being most dynamic during the preclinical disease phase,^7,30^ similar to CSF Aβ_42_ and p-tau.

In line with our hypotheses, the observed differences between models with binary or continuous biomarkers indicate that dichotomization in some cases may decrease sensitivity to predict changes in cognition. Moreover, permutation findings suggest that biomarkers that are dynamic at a given disease stage are less suited to binarization; among CU participants, for instance, *R*^2^ values decreased when binarizing amyloid PET, CSF p-tau, and NfL, but were relatively unaffected when tau PET and MRI-based measures were dichotomized. By contrast, dichotomization of tau PET and MRI-based measures decreased *R*^2^ values among CI participants. Though the use of quartiles was superior to binary data, our findings do not support them being an optimal alternative to continuous data. These findings require confirmation in follow-up studies with larger cohorts and more varied cognitive measures, but they point to the fact the binarization may lead to loss of information when the tested biomarker changes on a continuum (e.g., tau PET) rather than more clear transitions from one stage to another (e.g., CSF Aβ_42_). In practice, this means that more complex models that take continuous data into account may outperform simple binarization, for example when selecting participants likely to decline cognitively in a clinical trial.

The study has limitations. First, the sample size for the analysis of longitudinal cognition was modest, which may reduce the power to detect associations between cognition and certain AT(N) variants. The use of the MMSE may have precluded insight into domain-specific cognitive decline, including nonamnestic aspects, as well as neurobehavioral changes that may be the first symptom of AD pathophysiology during the preclinical phase of AD.^31–33^ Further, the use of the Braak V/VI ROI, reflecting widespread neocortical tau,^34^ was of greater relevance to the CI group, as neocortical tau pathology is almost invariably associated with cognitive impairment.^35^ Findings among CU participants, however, did not differ when using a region important for very early tau pathology (the entorhinal cortex)^34^ (i.e., T+ remained greater when using CSF p-tau compared to tau PET, and A2T1(N)2 remained the best predictor of longitudinal cognition). Finally, although this is, to our knowledge, the most comprehensive study to date testing different biomarkers for AT(N), we acknowledge that several other biomarkers could also be tested (e.g., [^18^F]FDG PET and novel CSF measures for tau and neurodegeneration).^36,37^

The AT(N) system is useful to classify participants, predict cognitive decline over time, and perhaps stratify participants for inclusion in clinical trials.^38^ The large variability in how participants are classified when using different biomarkers shows that different AT(N) variants are not interchangeable. Optimal biomarker combinations for diagnosis and prediction of rapid cognitive decline may vary by clinical stage. Moreover, dichotomizing some biomarkers results in the loss of important information compared to using them on continuous scales.

## Conflict of interest

N. Mattsson-Carlgren has served as a consultant for the Alzheimer's Disease Neuroimaging Initiative. A. Leuzy, S. Janelidze, S. Palmqvist, E. Stomrud, and O. Strandberg report no disclosures. Dr. Smith has served as a non-paid consultant for Roche. O. Hansson has acquired research support (for the institution) from Roche, GE Healthcare, Biogen, AVID Radiopharmaceuticals, and Euroimmun, and in the past 2 years, he has received consultancy/speaker fees (paid to the institution) from Biogen and Roche.

## Glossary

Aβ: β-amyloid
AD: Alzheimer disease
ADNI: Alzheimer's Disease Neuroimaging Initiative
ANOVA: analysis of variance
AT(N): β-amyloid, tau, and neurodegeneration classification system
CI: cognitively impaired
CU: cognitively unimpaired
DSM-5: *Diagnostic and Statistical Manual of Mental Disorders, 5th edition*
GMM: Gaussian mixture modeling
IQR: interquartile range
ITC: inferior temporal cortex
LME: linear mixed effects
MCI: mild cognitive impairment
MMSE: Mini-Mental State Examination
NfL: neurofilament light
p-tau: tau phosphorylated at Thr181
ROI: region of interest
SUVR: standardized uptake value ratio
